# Efeitos de Diferentes Tipos de Treinamento Físico na Função Endotelial em Pré-Hipertensos e Hipertensos: Uma Revisão Sistemática

**DOI:** 10.36660/abc.20190807

**Published:** 2021-05-06

**Authors:** Gustavo Waclawovsky, Marinei L. Pedralli, Bruna Eibel, Maximiliano I. Schaun, Alexandre M. Lehnen

**Affiliations:** 1 Fundação Universitária de Cardiologia Instituto de Cardiologia do Rio Grande do Sul Laboratório de Pesquisas Clínicas Porto AlegreRS Brasil Laboratório de Pesquisas Clínicas - Instituto de Cardiologia do Rio Grande do Sul / Fundação Universitária de Cardiologia, Porto Alegre, RS – Brasil

**Keywords:** Endotélio, Células Tronco, Exercício Físico, treinamento resistido, Hipertensão, Revisão

## Abstract

**Fundamento:**

A hipertensão sustentada pode levar ao remodelamento vascular e lesão das células endoteliais, o que pode explicar a disfunção endotelial encontrada em hipertensos. O treinamento físico pode melhorar a saúde vascular em indivíduos com risco cardiovascular, mas pouco se sabe sobre seus efeitos em pré-hipertensos e hipertensos.

**Objetivo:**

Revisar a literatura mostrando evidências de alterações da função endotelial em resposta a diferentes modalidades de treinamento físico em pré-hipertensos e hipertensos.

**Métodos:**

Realizamos uma revisão sistemática de estudos nas bases de dados MEDLINE, Cochrane, LILACS, EMBASE e SciELO seguindo tanto as diretrizes PRISMA (Preferred Reporting Items for Systematic Reviews and Meta-Analyzes) quanto a estratégia PICO (paciente/população, intervenção, comparação e resultados). Os ensaios clínicos randomizados (ECRs) publicados até abril de 2019 foram selecionados e avaliados por quatro revisores independentes. A qualidade metodológica foi avaliada por meio da escala PEDro (Physiotherapy Evidence Database).

**Resultados:**

Nossa busca rendeu 598 resumos, e 10 estudos foram elegíveis para revisão. Todos eles apresentaram qualidade metodológica aceitável pela escala PEDro. Dos 10 estudos, 7 envolveram treinamento aeróbico, 1 treinamento resistido isométrico e 2 treinamento aeróbico e treinamento resistido dinâmico separadamente. Sete estudos usaram dilatação fluxo-mediada (DFM) para avaliar a saúde vascular, e três usaram pletismografia. A maioria dos protocolos de treinamento envolveu indivíduos hipertensos e consistiu em exercícios de baixa e moderada intensidade.

**Conclusão:**

Nossa revisão sistemática mostrou que o treinamento aeróbico contínuo moderado é eficaz para melhorar a saúde vascular em indivíduos hipertensos. Em pré-hipertensos, o treinamento aeróbico intervalado vigoroso parece ser uma alternativa para benefícios à saúde vascular. O treinamento físico resistido isométrico ou dinâmico pode ser usado como alternativa secundária, mas ainda requer mais investigação.

## Introdução

**Figura 1:**
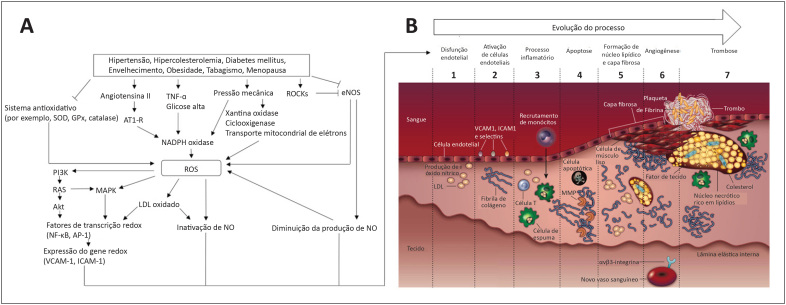
Integração geral das espécies reativas de oxigênio com aterosclerose e balanço da lesão endotelial versus recuperação. Painel A-B: Representação esquemática da geração de ROS induzida por respostas inflamatórias e vasoconstritoras em estados de doença e estilo de vida não saudável, bem como seus efeitos no processo de disfunção endotelial e formação de placa aterosclerótica. NO: óxido nítrico; ROCK: Rho-quinase associada; SOD, superóxido dismutase; AT1-R: Receptor AT1; NADH: Nicotinamida Adenina Dinucleotídeo reduzido; ROS: espécies reativas de oxigênio; eNOS: óxido nítrico sintase 3; PI3K: Fosfatidilinositol-4,5-bisfosfato 3-quinase; RAS: sistema renina-angiotensina; MAPK: proteína quinase ativada por mitogênio; Akt: proteína quinase B; NF-κB: fator nuclear kappa B; AP-1: Proteína ativadora 1; VCAM-1: molécula 1 de adesão de células vasculares; ICAM-1: Molécula de Adesão Intercelular 1; MMP: metaloproteinases de matriz. Adaptado de Higashi et al (2009) e Sanz e Fayad (2008).

A hipertensão arterial sistêmica é uma condição multifatorial caracterizada por níveis elevados de pressão arterial (PA) sustentada. Um aumento de 20 mmHg na pressão arterial sistólica (PAS) em indivíduos com idades entre 40 e 69 anos tem sido associado a um risco duas vezes maior de morte por doença isquêmica do coração devido a doença vascular.^1^ Eventos cardiovasculares estão intimamente relacionados à disfunção vascular, em particular devido ao comprometimento da função do tecido endotelial, que desempenha um papel central na regulação do tônus vascular e da resistência vascular periférica.^2^ Função endotelial prejudicada, níveis elevados de micropartículas endoteliais circulantes (MPE)^3^ e menor capacidade regenerativa vascular, caracterizada pela redução da mobilização de células progenitoras endoteliais (CPE),^4^^,^^5^ já estão bem descritos em hipertensos e são a principal causa de aterosclerose e consequentes eventos cardiovasculares fatais e não fatais nessa população^6^ (Figura 1).

Mudanças no estilo de vida, como a inclusão de prática de atividade física regular, são recomendadas como abordagem terapêutica para restaurar a função endotelial em indivíduos com hipertensão.^7^^,^^8^ Os exatos mecanismos subjacentes aos potenciais efeitos anti-hipertensivos e a resposta endotelial de longo prazo ao exercício ainda não são totalmente compreendidos, mas uma redução na atividade simpática,^9^ um equilíbrio entre vasodilatadores e vasoconstritores^10^ e uma redução nos níveis do vasoconstritor endotelina-1 (ET-1)^11^ já foram investigados.

O exercício aeróbico regular pode prevenir a perda da vasodilatação dependente do endotélio até mesmo em idosos.^12^ Este benefício está associado ao aumento do estresse de cisalhamento nas paredes vasculares em reposta ao exercício físico. Portanto, o exercício regular aumenta a produção de óxido nítrico (NO), aumenta a expressão de óxido nítrico sintase e dilata todos os tipos de vasos sanguíneos, estimulando a guanilato ciclase solúvel e aumentando o monofosfato cíclico de guanosina (GMPc) nas células musculares lisas. Também promove angiogênese por meio do fator de crescimento endotelial vascular e aumenta a resposta antioxidante local, que por sua vez preserva a biodisponibilidade do óxido nítrico endotelial.^13^

Uma metanálise de estudos de indivíduos com vários fatores de risco cardiovascular e/ou doença cardiovascular estabelecida demonstrou que o treinamento aeróbico e resistido podem melhorar a resposta à dilatação dependente do endotélio.^14^ Outros estudos relatam os benefícios do exercício regular por promover a expressão de moléculas de adesão, modulação da resposta inflamatória^15^ e mobilização de CPE.^16^ Ainda assim, esse corpo de evidências provém de estudos conduzidos com populações altamente heterogêneas, o que dificulta conclusões para a população particular de indivíduos pré-hipertensos e hipertensos.

**Figura 2:**
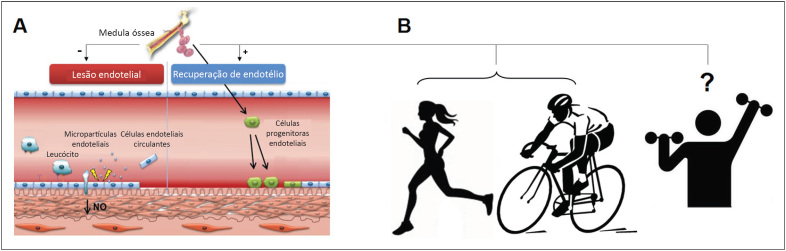
Painel A-B: Hipótese do processo adaptativo modulado pelo treinamento físico para restaurar o equilíbrio dano/reparo do tecido endotelial e a manutenção de sua função vasomotora.

De fato, nosso grupo publicou uma metanálise abordando os efeitos do treinamento físico na função endotelial.^17^ No entanto, apenas exercícios aeróbicos foram incluídos e o resultado foi avaliado por dilatação fluxo-mediada (DFM). Assim, a presente revisão sistemática tem um escopo mais amplo, pois discute os mecanismos potenciais envolvidos na associação entre o treinamento físico e a função endotelial (Figura 2). Assim, semelhante à técnica de DFM, a pletismografia é fortemente dependente do NO endotelial^18^^–^^20^ e, portanto, ambas as técnicas são amplamente utilizadas quando a função endotelial é o desfecho de interesse. Optamos por incluir pletismografia e exercícios resistidos, que não foram abordados em nossa metanálise anterior. Assim, realizamos uma revisão sistemática de estudos que mostram evidências das alterações da função endotelial em resposta a diferentes modalidades de treinamento físico em pré-hipertensos e hipertensos. Em seguida, examinamos as evidências sobre marcadores endoteliais, como mobilização de CPE e MPE.

## Materiais e métodos

### Seleção de estudos

Esta revisão sistemática seguiu as diretrizes PRISMA (Preferred Reporting Items for Systematic Reviews and Meta-Analyzes)^21^ e foi conduzida até abril de 2019 por quatro revisores independentes (G.W., M.I.S., M.L.P. e B.E.) nas seguintes bases de dados: MEDLINE (acessado via PubMed), Cochrane Central Register of Controlled Trials (Cochrane); Centro Latino-Americano e do Caribe de Informação em Ciências da Saúde (LILACS); EMBASE e Scientific Electronic Library Online (SciELO). Não estabelecemos limites para a data de publicação, e artigos em português, inglês ou espanhol eram elegíveis para inclusão.

**Figura 3:**
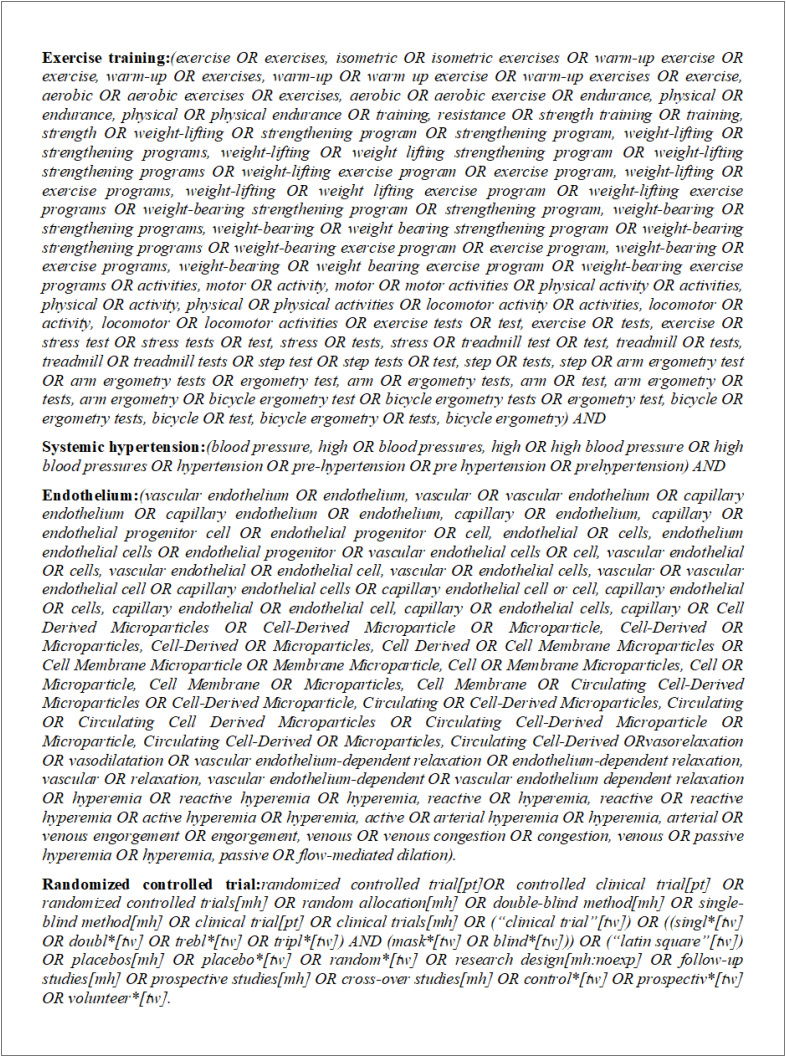
Descritores MeSH (Medical Subject Headings for PubMed) para busca no MEDLINE.

O conjunto de termos *exercise, systemic hypertension* e *endothelium* foi utilizado para a busca de estudos nas bases de dados Cochrane, LILACS, EMBASE e SciELO. Para a pesquisa MEDLINE, usamos três conjuntos diferentes de descritores MeSH (Figura 3). Para aumentar a precisão e a sensibilidade de nossa pesquisa de desenhos de estudo (*Randomized Controlled Trial*, RCTs) no banco de dados MEDLINE, adicionamos os termos de pesquisa para RCTs (Figura 3).^22^ Além disso, utilizamos a estratégia PICO^21^ (paciente/população, intervenção, comparação e desfechos) para inclusão dos estudos.

Os quatro revisores realizaram a seleção dos estudos e revisaram independentemente os títulos e resumos. Quando os resumos não forneciam informações suficientes, eles realizavam uma leitura completa dos artigos. Os revisores resolveram quaisquer discrepâncias por consenso; quaisquer discordâncias sobre os critérios de inclusão foram resolvidas por outro revisor (A.M.L.). As informações sobre o número de artigos envolvendo treinamento aeróbico, resistido e combinado, bem como as intensidades dos exercícios e técnicas utilizadas para medir a função endotelial, foram definidas por grupos: pré-hipertensão e hipertensão.

### Critérios de inclusão e exclusão

Os critérios de inclusão foram: (a) adultos com 18 anos ou mais; (b) indivíduos com pré-hipertensão ou hipertensão sistêmica; (c) treino regular de exercícios como parte do protocolo de intervenção; (d) mobilização de CPE ou MPE como resultado do estudo; (e) avaliação endotelial por DFM ou pletismografia, número de CPE avaliado por citometria de fluxo ou cultura de células e número de MPE avaliados por citometria de fluxo.

Foram excluídos estudos sobre intervenções medicamentosas, intervenções dietéticas ou uma única sessão de exercícios, bem como estudos envolvendo animais, crianças/adolescentes e apenas indivíduos normotensos, ensaios clínicos não randomizados e publicações duplicadas. Também foram excluídos estudos cujos participantes tinham doenças metabólicas e outras doenças cardiovasculares além da hipertensão.

A avaliação da qualidade dos estudos foi baseada na escala Physiotherapy Evidence Database (PEDro)^23^ (Tabela 1S, material suplementar).

### Análise estatística

Todos os dados foram tabulados como variáveis categóricas no Microsoft Excel e uma análise descritiva no SPSS for Windows, versão 24 (Chicago, IL), foi realizada por um dos pesquisadores (G.W.).

## Resultados

**Figura 4:**
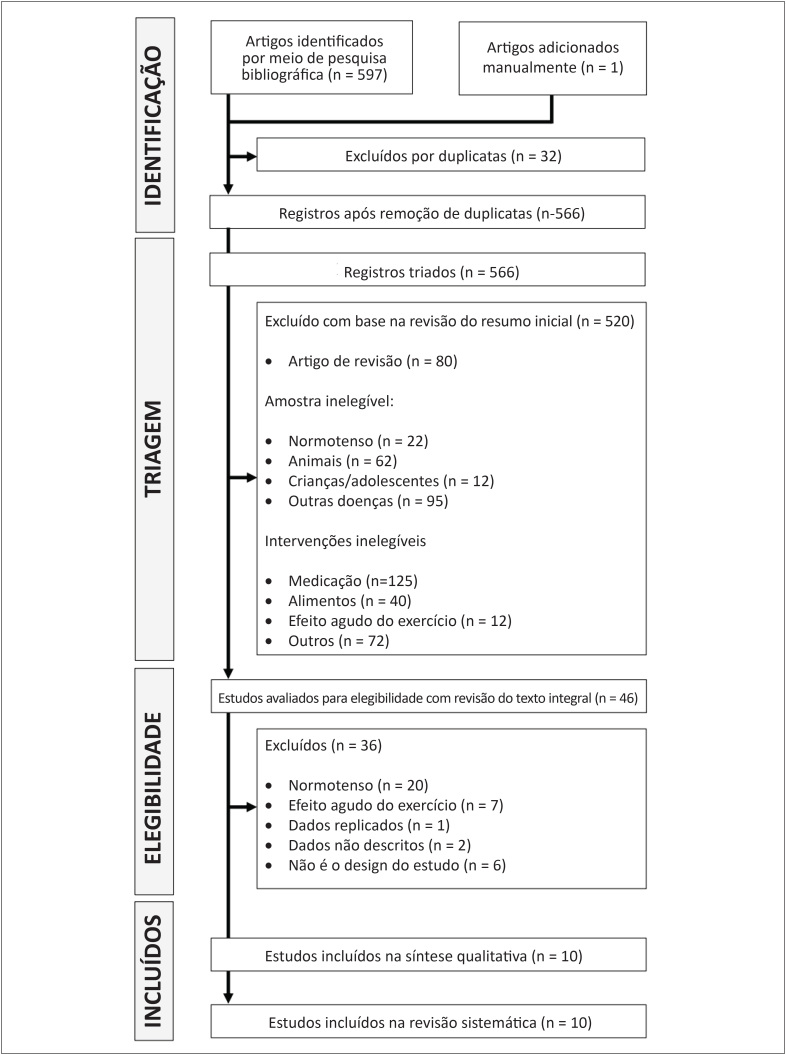
Fluxograma da seleção dos ensaios clínicos randomizados incluídos nesta revisão sistemática.

Esta revisão sistemática teve como objetivo avaliar as evidências de alterações na função endotelial em resposta ao treinamento físico aeróbico, resistido e combinado (pré- vs. pós-treinamento) em indivíduos pré-hipertensos e hipertensos. Encontramos em nossa busca 598 resumos (297 na MEDLINE; 43 na Cochrane; 47 na LILACS; 200 na EMBASE; e 11 na SciELO). Todos os títulos e resumos foram revisados e, em seguida, 46 artigos foram lidos na íntegra e revisados para checagem de elegibilidade. Dez artigos foram selecionados para revisão (Figura 4).

Dentre os dez estudos, quatro somaram 7 pontos, outros quatro somaram 6 pontos e apenas dois estudos somaram 5 pontos na escala PEDro. No entanto, é importante destacar que a abordagem cega (treinamento físico) não foi fornecida, pois não é aplicável neste tipo de intervenção. Assim, consideramos todos os estudos incluídos de qualidade aceitável de acordo com a escala PEDro.

A Tabela 2S (material suplementar) mostra informações detalhadas dos estudos revisados. Resumidamente, dos dez estudos selecionados, sete envolveram treinamento aeróbico, um abordou o treinamento resistido isométrico, dois o treinamento aeróbico e o treinamento resistido dinâmico separadamente e nenhum envolveu a combinação de exercícios aeróbicos e resistido na mesma sessão (treinamento combinado). Destes, apenas três estudos compararam os efeitos de diferentes tipos de treinamento físico na função endotelial (treinamento contínuo e intervalado; resistido dinâmica e treinamento intervalado). O tamanho da amostra variou de 16 a 155, totalizando 519 pré-hipertensos e hipertensos.

Dos estudos selecionados, as amostras de sangue foram analisadas em busca de marcadores de saúde vascular em apenas dois deles. CPE e MPE não foram avaliados em nenhum dos RCTs com pré-hipertensos ou hipertensos. A função vasomotora endotelial foi avaliada por DFM (dilatação fluxo-mediada da artéria braquial avaliada por ultrassom) em sete estudos e pletismografia (vasodilatação total do antebraço ou panturrilha capturada por medições de fluxo por um extensor de elástico local) em três estudos (descrição detalhada da pletismografia pode ser vista em Bystrom et al.^24^ e Waclawovsky et al.^25^).

De acordo com a classificação de intensidade de exercício do American College of Sports Medicine,^26^ o treinamento aeróbico de baixa intensidade foi avaliado em três estudos, o exercício moderado em quatro estudos e o exercício vigoroso em três estudos. Para o treinamento de resistência isométrica, selecionamos apenas um estudo de baixa intensidade. Para o treinamento de resistência dinâmico, a intensidade moderada foi examinada em dois estudos, enquanto o treinamento de intensidades baixa e vigorosa não foi avaliado em nenhum deles.

A duração do treinamento físico foi na maioria das vezes de 12 semanas (seis estudos), seguida de 8 semanas (três estudos) e 24 semanas (um estudo). O número de sessões de variou: 3 vezes por semana (sete estudos); 4 vezes por semana (um estudo); e 5 ou mais vezes por semana (dois estudos).

## Discussão

Esta revisão sistemática teve como objetivo avaliar quaisquer evidências de alterações na função endotelial em resposta a diferentes modalidades de treinamento físico em indivíduos pré-hipertensos e hipertensos. Os resultados mostraram que o treinamento aeróbico contínuo em intensidade moderada (50% VO_2_máx), por 30-40 minutos por sessão pelo menos 3 vezes por semana, parece ser a intervenção mais adequada para melhorar a vasodilatação endotélio-dependente em hipertensos. Em pré-hipertensos, o treinamento aeróbico intervalado vigoroso (3 min/caminhada e 2 min/corrida; 65% e 85% da FCmáx), em sessões de 45 minutos, 3 vezes por semana parece ser uma alternativa para beneficiar a saúde vascular. Em relação ao treinamento resistido, encontramos um estudo relatando os efeitos do treinamento isométrico de baixa intensidade (30% do esforço máximo, 4 vezes por 2 minutos, 3 vezes por semana) na função endotelial em indivíduos hipertensos; e o treinamento resistido de intensidade moderada (8 exercícios, 60 min/sessão, 3 vezes por semana, 2×8-12 repetições até a fadiga muscular local) foi examinado em dois estudos conduzidos com indivíduos pré-hipertensos. Com base nos ensaios clínicos randomizados envolvendo treinamento isométrico avaliado, podemos especular que o treinamento de baixa intensidade pode melhorar a função endotelial localizada. Já o treinamento resistido dinâmico pode ser uma alternativa para melhorar a função endotelial em pré-hipertensos quando realizado em intensidade moderada.

### Treinamento aeróbico

Westhoff et al.,^27^ desenvolveu um protocolo de treinamento de exercício aeróbico de baixa intensidade (sessões 2 vezes por semana durante 12 semanas) usando um cicloergômetro de membros superiores para avaliar a resposta vascular em pacientes com hipertensão e não encontrou melhora na vasodilatação dependente do endotélio dos vasos do braço. Uma explicação pode ser a intensidade do exercício: o lactato sanguíneo era 2,0 mmol/L, que é um nível próximo ao de repouso. A associação com a intensidade do exercício é ainda suportada pelo fato de que os indivíduos com hipertensão estágio I-II apresentaram melhora da vasodilatação endotélio-dependente dos vasos do braço após treinamento aeróbico em exercícios de intensidade quase máxima.^28^ Além do exercício de baixa intensidade neste estudo, o uso de betabloqueadores pode ter causado diminuição do volume sistólico e do débito cardíaco e redução da liberação de NO mediada pelo estresse de cisalhamento das células endoteliais, causando, portanto, menor vasodilatação.^29^

A intensidade do exercício aeróbico parece influenciar a resposta vasomotora em indivíduos hipertensos. O treinamento aeróbico por uma hora em bicicleta ergométrica, 3 vezes por semana por 6 meses em intensidade moderada (50% FCreserva), demonstrou aumentar os níveis plasmáticos de NO em mulheres hipertensas.^30^ Molmen-Hansen et al.^28^ relatou que um treinamento aeróbico de 3 meses melhorou a vasodilatação endotélio-dependente dos vasos do braço em indivíduos hipertensos apenas em altas intensidades (exercícios alternados a 60-70% e 90-95% da FCmáx). Levanta-se a questão de quais outros fatores além do aumento dos níveis de NO poderiam contribuir para a melhora da função vasomotora em resposta ao treinamento aeróbico em indivíduos hipertensos.

A hipertensão arterial está associada ao aumento da atividade simpática, que é intensificada durante o exercício. Em indivíduos normotensos, o exercício causa uma atenuação da atividade simpática nos músculos ativos com consequente vasodilatação local.^31^ Essa vasodilatação local tardia é paralela ao aumento da intensidade do trabalho muscular, e esse fenômeno envolve alterações nos metabólitos musculares e outras substâncias para reduzir a resposta vascular à ativação de receptores α-adrenérgicos envolvidos na regulação do tônus vascular.^32^ Por outro lado, esse mecanismo é atenuado em indivíduos hipertensos^31^ e, junto com o aumento da rigidez arterial, leva à redução do fluxo sanguíneo e menor estresse de cisalhamento durante o exercício.^33^ Esses fatores podem atuar em conjunto, impossibilitando a melhora na capacidade vasomotora em hipertensos após exercício aeróbico em intensidade moderada ou próxima a moderada, mesmo com síntese de NO preservada.^30^

O treinamento intervalado parece ser benéfico para a saúde vascular de indivíduos pré-hipertensos. Conforme demonstrado por Beck et al.,^34^ um programa de treinamento de exercícios que consiste em caminhar por 3 minutos em intensidade moderada, alternando com corrida por 2 minutos em intensidade vigorosa (exercícios alternados em 65-85% da FCmáx), 3 vezes por semana e durante 8 semanas pode aumentar a vasodilatação dependente do endotélio em jovens pré-hipertensos.^35^

Ao contrário do corpo de evidências sobre exercícios de alta intensidade, alguns estudos relataram melhorias na vasodilatação dependente do endotélio dos vasos do braço em pacientes idosos hipertensos após 12 semanas de treinamento aeróbico de baixa intensidade (nível de lactato sanguíneo ≤2,5 mmol/L).^36^^,^^37^ No entanto, um fator importante que afeta a melhora da função endotelial após exercício aeróbico em indivíduos com hipertensão é a disfunção endotelial, ou seja, vasodilatação endotélio-dependente avaliada por DFM menor que 5,5%.^38^ Assim, a variação dos resultados de estudos envolvendo exercícios aeróbicos em intensidade baixa e moderada também pode ser explicada pela disfunção endotelial basal dos participantes.

A saúde vascular em hipertensos em resposta ao treinamento aeróbico pode ser influenciada pelo perfil lipídico. Em dois estudos, Higashi et al.,^39^^,^^40^ demonstraram que um treinamento de 3 meses, consistindo em caminhada não supervisionada 5–7 vezes por semana em intensidade moderada (50% VO_2_máx) por 30 minutos, melhorou a vasodilatação dos vasos do antebraço em indivíduos hipertensos não tratados.^39^^,^^40^ Curiosamente, a melhora na vasodilatação dos vasos do antebraço foi negativamente correlacionada com os níveis de colesterol LDL. Assim, como a hipertensão está comumente associada a níveis baixos de HDL e níveis elevados de LDL, a não modificação do perfil lipídico nessa população pode contribuir para uma melhoria insatisfatória da função endotelial.

Os níveis circulantes de MPE no sangue periférico estão associados à integridade endotelial. MPE são pequenas vesículas de membrana liberadas das células endoteliais em resposta à ativação, lesão e apoptose celular. Os principais marcadores de superfície celular incluem CD144+, CD31+/CD41-, CD31+/CD42b-, CD31+/anexina V+ e CD62E.^41^ Os MPE têm sido associados ao escore de risco de Framingham,^3^ hipertensão,^42^ entre outras condições. Feairheller et al.,^43^ avaliaram, em afroamericanos, os efeitos do treinamento aeróbico vigoroso (até 65% do VO_2_máx) por 6 meses.^43^ Eles relataram que a DFM aumentou 60% e os níveis plasmáticos de NO aumentaram 77%, junto com uma redução de 50% nas contagens de MPE. Porém, dos 25 indivíduos da amostra, 10 eram normotensos, 9 pré-hipertensivos e apenas 7 eram hipertensos, dificultando a extrapolação dos dados para as três populações. Parece que o estresse de cisalhamento induzido pelo exercício pode preservar a função endotelial por meio de um mecanismo que potencializa as funções metabólicas das células vasculares.

O equilíbrio entre lesão endotelial e reparo é o evento mais significativo na patogênese da aterosclerose. Os CPE desempenham um papel importante na reparação de células endoteliais lesadas e na manutenção da integridade endotelial. Um baixo número de CPE expressando o fenótipo CD34+/KDR+ é preditivo de eventos cardiovasculares e morte,^44^ e baixos níveis de CPE expressando o fenótipo CD34+/KDR+/CD45dim é um forte preditor de progressão da doença aterosclerótica.^4^ Está bem estabelecido que os indivíduos hipertensos apresentam baixo número de CPE funcionais.^5^ Por sua vez, o treinamento aeróbico aumenta os níveis de CPE em pacientes com risco cardiovascular ou doença cardiovascular estabelecida,^45^ equilibrando lesão e reparo endotelial. No entanto, não encontramos estudos associando treinamento aeróbio e CPE em pré-hipertensos e hipertensos. Mais investigações sobre esse tema são necessárias.

### Treinamento resistido

Até o momento, apenas um RCT relatou os resultados do treinamento isométrico sobre a função endotelial em hipertensos. Os autores avaliaram a vasodilatação endotélio-dependente dos vasos do braço em indivíduos hipertensos após treinamento de preensão manual isométrica unilateral e bilateral.^46^ Curiosamente, a vasodilatação dependente do endotélio melhorou apenas no braço treinado (o DFM do braço treinado aumentou de 2,4 para 6,6%, p<0,001; sem alteração observada no braço não treinado).^46^ Portanto, pode-se supor que uma maior massa muscular submetida ao treinamento seja necessária para alcançar benefícios globais da função endotelial nessa população. É importante ressaltar que a maioria dos artigos incluídos realizava a técnica DFM de Corretti et al.,^47^ mesmo os publicados após 2011, período em que a técnica já havia sido atualizada. Isso nos permite questionar como a técnica atual, mais precisa, poderia alterar os resultados vasculares encontrados, otimizando-os.

Beck et al.,^34^ examinaram os efeitos do treinamento resistido em indivíduos pré-hipertensos e descobriram que o treinamento de uma hora, 3 vezes por semana durante 2 meses, consistindo de 2×8 a 12 repetições máximas (intensidade moderada), aumentou a vasodilatação dependente do endotélio dos vasos do braço e reduziu os níveis de ET-1. Esse mesmo protocolo foi repetido para avaliação da função vascular em membros superiores e inferiores por pletismografia de oclusão venosa.^35^ Foi reportada uma melhora na vasodilatação dos vasos do antebraço e das pernas, bem como maior equilíbrio oxidante-antioxidante ao final de dois meses de treinamento. O aumento da vasodilatação endotélio-dependente pode ser explicado pela oclusão mecânica dos vasos durante o exercício, que causa períodos contínuos de isquemia e reperfusão nos membros treinados, aumenta o o estresse de cisalhamento e leva a alterações adaptativas endoteliais locais que aumentam cronicamente a capacidade vasodilatadora.^33^ Outra explicação possível é o aumento do fluxo sanguíneo para os músculos treinados. Essa redistribuição do fluxo sanguíneo durante o exercício aumenta o fluxo sanguíneo sistólico anterógrado e diastólico retrógrado, que pode induzir aumento do estresse de cisalhamento nos vasos dos membros destreinados.^29^

Ao contrário do exercício resistido, o exercício aeróbico aumenta continuamente o fluxo sanguíneo, o que pode levar a um aumento no estresse de cisalhamento^48^ e maiores adaptações vasculares induzidas pelo exercício quando comparado a outras modalidades. No entanto, melhorias na função endotelial em membros não treinados parecem ser semelhantes em indivíduos jovens saudáveis e indivíduos com diabetes tipo 1 após uma sessão de exercícios aeróbicos e resistido com duração, intensidade e grupos musculares treinados semelhantes.^25^ Esse achado levanta a possibilidade de que essas variáveis possam ter impactado os resultados e possam explicar inconsistências entre os estudos.

Não encontramos estudos envolvendo treinamento isométrico isométrico ou dinâmico que medissem MPE e CPE em pré-hipertensos e hipertensos. Requer mais investigação.

Nosso estudo apresenta algumas limitações que precisam ser consideradas. Diferentes estratégias de treinamento aeróbico (caminhada rápida, ciclismo e esteira), idades dos participantes e tempos de intervenção avaliados nos RCTs dificultam a inferência do efeito de cada fator na função endotelial. Dado o corpo limitado de evidências para o treinamento resistido, mais investigações são necessárias para que possamos delinear os efeitos da melhora na função endotelial em indivíduos com pressão arterial alterada, uma vez que até agora são especulativos.

## Conclusão

Nos estudos incluídos em nossa revisão sistemática, o treinamento aeróbico de intensidade moderada por 30–40 minutos/sessão e pelo menos 3 vezes por semana é eficaz para melhorar a função endotelial em indivíduos hipertensos. Em pré-hipertensos, o treinamento aeróbico intervalado de intensidade vigorosa, 45 minutos/sessão e 3 vezes por semana parece ser uma alternativa para trazer benefícios à saúde vascular. Em perspectiva, o treinamento resistido, seja isométrico ou dinâmico, pode ser utilizado como estratégia secundária para melhorar a função endotelial de indivíduos com medidas alteradas da pressão arterial. Com relação aos dados de CPE e MPE, nenhum estudo envolvendo treinamento de força isométrico ou dinâmico mediu MPE e CPE em pré-hipertensos e hipertensos.
