# Supplementary material for: Efeitos de Diferentes Tipos de Treinamento Físico na Função Endotelial em Pré-Hipertensos e Hipertensos: Uma Revisão Sistemática
Source: Arq Bras Cardiol. 2021 May 6;116(5):938–47. [Article in Portuguese] doi: 10.36660/abc.20190807 (PMC8121478; doi:10.36660/abc.20190807)
Supplement: Translation

# Translation: Effects of Different Types of Exercise Training on Endothelial Function in Prehypertensive and Hypertensive Individuals: A Systematic Review

**Authors:** Gustavo Waclawovsky, Marinei L. Pedralli, Bruna Eibel, Maximiliano I. Schaun, Alexandre M. Lehnen

**Affiliations:** 1 Porto AlegreRS Brasil Laboratório de Pesquisas Clínicas - Instituto de Cardiologia do Rio Grande do Sul / Fundação Universitária de Cardiologia, Porto Alegre, RS – Brasil

**Keywords:** Endothelium, Stem Cells, Exercise, Resistance Training, Hypertension, Review

## Abstract

**Background:**

Sustained high blood pressure can lead to vascular remodeling and endothelial cell injury, which may explain the endothelial dysfunction found in hypertensive individuals. Exercise training can improve vascular health in individuals with cardiovascular risk, but little is known about its effects in prehypertensive and hypertensive individuals.

**Objective:**

To review the literature showing evidence of changes in endothelial function in response to different modalities of exercise training in prehypertensive and hypertensive individuals.

**Methods:**

We conducted a systematic review of studies in the MEDLINE, Cochrane, LILACS, EMBASE, and SciELO databases following both the PRISMA guidelines (Preferred Reporting Items for Systematic Reviews and Meta-Analyses) and the PICO framework (patient/population, intervention, comparison and outcomes). Randomized clinical trials (RCTs) published up to April 2019 were selected and assessed by four independent reviewers. The methodological quality was assessed using the PEDro (Physiotherapy Evidence Database) scale.

**Results:**

Our search yielded 598 abstracts, and 10 studies were eligible for review. All of them had acceptable methodological quality by PEDro scale. Of the 10 studies, 7 involved aerobic training, 1 isometric resistance training, and 2 aerobic training and dynamic resistance training separately. Seven studies used flow-mediated dilation (FMD) to assess the vascular health, and three used plethysmography. Most training protocols involved hypertensive individuals and consisted of low and moderate-intensity exercise.

**Conclusion:**

Our systematic review showed that moderate continuous aerobic training is effective to improve vascular health in hypertensive individuals. In prehypertensive individuals, vigorous interval aerobic training seems to be an alternative to determine vascular health benefits. Resistance exercise training, either isometric or dynamic, can be used as a secondary alternative, but still requires further investigation.

## Introduction

Systemic hypertension is a multifactorial condition characterized by sustained high blood pressure (BP) levels. An increase of 20 mmHg in systolic blood pressure (SBP) in individuals at the ages of 40–69 years has been associated with a risk 2 times higher of death from ischemic heart disease, due to vascular disease.^1^ Cardiovascular events are closely related to vascular dysfunction, in particular due to the impaired endothelial tissue function which plays a central role in the regulation of vascular tone and peripheral vascular resistance.^2^ Impaired endothelial function, high levels of circulating endothelial microparticles (EMP)^3^ and a lower vascular regenerative capacity, characterized by reduced mobilization of endothelial progenitor cells (EPC),^4^^,^^5^ is well described in hypertensive individuals and is the main cause of atherosclerosis and consequent fatal and nonfatal cardiovascular events in this population^6^ (Figure 1).

**Figure 1:** General integration of reactive oxygen species with atherosclerosis and balance of endothelial injury versus recovery. Panel A-B: Schematic representation of ROS generation induced by inflammatory and vasoconstrictor responses in disease states and unhealthy lifestyle, and its effects on the process of endothelial dysfunction and atherosclerotic plaque formation. NO: nitric oxide; ROCK: Rho kinase associated; SOD, superoxide dismutase; AT1-R: Receptor AT1; NADPH: reduced nicotinamide adenine dinucleotide phosphate; ROS: reactive oxygen species; eNOS: nitric oxide synthase 3; PI3K: Phosphatidylinositol-4,5-bisphosphate 3-kinase; RAS: renin–angiotensin system; MAPK: mitogen-activated protein kinase; Akt: Protein kinase B; NF-κB: nuclear factor kappa B; AP-1: Activator protein 1; VCAM-1: vascular cell adhesion molecule 1; ICAM-1: Intercellular Adhesion Molecule 1; MMP: matrix metalloproteinases. Adapted from Higashi et al (2009) and Sanz and Fayad (2008).

Lifestyle changes such as regular physical activity are recommended as a therapeutic approach for restoring endothelial function in individuals with hypertension.^7^^,^^8^ The exact mechanisms underlying the potential antihypertensive effects and long-term endothelial response to exercise are not fully understood, but a reduction in sympathetic activity,^9^ a balance between vasodilators and vasoconstrictors^10^ and a reduction in the levels of the vasoconstrictor endothelin-1 (ET-1)^11^ have been investigated.

Regular aerobic exercise may prevent the loss of endothelium-dependent vasodilation, even in elderly individuals.^12^ This benefit is associated with exercise-induced increases in shear stress on vascular walls. Therefore, regular exercise increases nitric oxide production, induces increased expression of nitric oxide synthase and dilates all types of blood vessels by stimulating soluble guanylate cyclase and increasing cyclic guanosine monophosphate in smooth muscle cells. It also promotes angiogenesis via the vascular endothelial growth factor and induces increased local antioxidant response, which in turn preserves endothelial nitric oxide bioavailability.^13^

A meta-analysis of individuals with several cardiovascular risk factors and/or established cardiovascular disease has demonstrated that aerobic and resistance exercise training can potentially improve endothelium-dependent dilation response.^14^ Other studies have reported the benefits of regular exercise because it promotes the expression of adhesion molecules, modulation of the inflammatory response^15^ and EPC mobilization.^16^ Yet, this body of evidence comes from studies conducted with highly heterogeneous populations, making it difficult to draw conclusions for the particular population of prehypertensive and hypertensive individuals.

Indeed, our group has published a meta-analysis addressing the effects of exercise training on endothelial function.^17^ However, only aerobic exercises were included and the outcome was evaluated by flow-mediated dilation (FMD). Thus, the present systematic review has a broader scope as it discusses potential mechanisms involved in the association between exercise training and endothelial function (Figure 2). Given that, similar to the FMD technique, plethysmography is strongly dependent on endothelial nitric oxide^18^^–^^20^ and, therefore, both techniques are widely used when endothelial function is the outcome of interest. We chose to include plethysmography and resistance exercises that were not addressed in our previous meta-analysis. Thus, we conducted a systematic review of studies showing evidence of the changes in endothelial function in response to different modalities of exercise training in prehypertensive and hypertensive individuals. Then, we examined the evidence on endothelial markers such as EPC mobilization and EMPs.

**Figure 2:** Panel A-B: Hypothesis of the adaptative process modulated by physical training to restore the damage/repair balance of the endothelial tissue and the maintenance of its vasomotor function.

## Materials and methods

### Selection of studies

This systematic review followed the PRISMA guidelines (Preferred Reporting Items for Systematic Reviews and Meta-Analyses)^21^ and it was conducted until April of 2019 by four independent reviewers (G.W., M.I.S. and M.L.P. and B.E.) on the following databases: MEDLINE (accessed via PubMed), Cochrane Central Register of Controlled Trials (Cochrane); *Latin American and Caribbean* Center on *Health Sciences* Information (LILACS); EMBASE and Scientific Electronic Library Online (SciELO). We set no publication date limits and articles in Portuguese, English or Spanish were eligible for inclusion.

The set of search terms *exercise, systemic hypertension* and *endothelium* was used to searching for studies in the Cochrane, LILACS, EMBASE and SciELO databases. For MEDLINE search, we used three different sets of MeSH descriptors (Figure 3). To increase the precision and the sensitivity of our search of study designs (Randomized Controlled Trial, RCTs) in MEDLINE database, we added the search terms for RCTs (Figure 3).^22^ Besides, we used the PICO strategy^21^ (patient/population, intervention, comparison and outcomes) for inclusion of studies.

**Figure 3:** MeSH (Medical Subject Headings for PubMed) descriptors for MEDLINE search.

The four reviewers undertook the selection of the studies, and independently reviewed titles and abstracts. When abstracts did not provide sufficient information, they performed a full reading of the articles. Reviewers solved any *discrepancies by consensus; any disagreements on* the inclusion criteria were settled by other reviewer (A.M.L.). Information about the number of articles involving aerobic, resistance and combined training as well as exercise intensities and techniques used to measure endothelial function were set by prehypertension and hypertension groups.

### Inclusion and exclusion criteria

The inclusion criteria were: (a) adults aged 18 or more; (b) individuals with prehypertension or systemic hypertension; (c) regular exercise training as part of the intervention protocol; (d) mobilization of EPCs or EMP counts as study outcomes; (e) endothelial assessment by FMD or plethysmography, number of EPCs measured by flow cytometry or cell culture and number of EMPs measured by flow cytometry.

Studies on drug interventions, dietary interventions or a single exercise session were excluded, as well as studies involving animals, children/adolescents, and only normotensive individuals; non-randomized clinical trials; duplicate publications. Studies with individuals with metabolic diseases and cardiovascular diseases other than hypertension were also excluded.

Quality assessment of the studies was based on the Physiotherapy Evidence Database (PEDro) scale^23^ (Table 1S, supplemental material).

### Statistical analysis

All data were tabulated as categorical variables in Microsoft Excel and a descriptive analysis using SPSS for Windows, version 24 (Chicago, IL), was performed by one of the investigators (G.W.).

## Results

This systematic review aimed to evaluate any evidence of changes in endothelial function in response to aerobic, resistance and combined exercise training (pre- vs. post-training) in prehypertensive and hypertensive individuals. We found in our search 598 abstracts (297 in MEDLINE; 43 in Cochrane; 47 in LILACS; 200 in EMBASE; and 11 in SciELO). All titles and abstracts were reviewed and then 46 articles were fully read and reviewed for their eligibility. Ten articles were selected for review (Figure 4).

**Figure 4:** Flowchart of the selection of randomized controlled trials included in this systematic review.

Among the ten studies, four scored 7 points, other four scored 6 points and only two studies scored 5 points in PEDro scale. However, it is important to highlight that the blinding intervention (exercise training) was not provided because it is not applicable in this kind of intervention. Thus, we considered all the included studies as of acceptable quality according to PEDro scale.

Table 2S (supplemental material) shows detailed information of the studies reviewed. Briefly, of the ten studies selected, seven involved aerobic training, one addressed isometric resistance training, two aerobic training and dynamic resistance training separately, and none involved the combination of aerobic and resistance exercise in the same session (combined training). Of these, only three studies compared the effects of different types of exercise training on endothelial function (continuous *versus* interval training; dynamic resistance *versus* interval training). The sample size ranged from 16 to 155, for a total of 519 prehypertensive and hypertensive individuals.

Of the studies selected, blood samples were analyzed for markers of vascular health in only two of them. EPCs and EMP were not measured in any RCTs with prehypertensive or hypertensive individuals. Endothelial vasomotor function was assessed by FMD (flow-mediated dilation of the brachial artery assessed by ultrasound) in seven studies and plethysmography (total vasodilation of the forearm or calf captured by local strain-gauge flow measurements) in three studies (a detailed description of plethysmography can be seen in Bystrom et al.^24^ and Waclawovsky et al.^25^).

According to the American College of Sports Medicine's exercise intensity classification,^26^ low-intensity aerobic training was examined in three studies, moderate exercise in four studies and vigorous exercise in three studies. For isometric resistance training, we selected only one study of low-intensity isometric training. For dynamic resistance training, moderate intensity was examined in two studies, while low and vigorous training in none of them.

The duration of exercise training was most often 12 weeks (six studies), followed by 8 weeks (three studies), and 24 weeks (one study). The number of training sessions varied: 3 times a week (seven studies); 4 times a week (one studies); and 5 or more times a week (two studies).

## Discussion

This systematic review aimed to evaluate any evidence of changes in endothelial function in response to different modalities of exercise training in prehypertensive and hypertensive individuals. The results showed that continuous aerobic training at moderate intensity (50% VO_2_max), for 30-40 minutes per session at least 3 times a week appears to be the most appropriate intervention to improve endothelium-dependent vasodilation in hypertensive individuals. In prehypertensive individuals, vigorous aerobic interval training (3 min/walking and 2 min/running; 65% and 85% HRmax), 45 minutes per session 3 times a week seems to be an alternative to determine vascular health benefits. Regarding resistance training, we found an RCT reporting the effects of low-intensity isometric resistance training (30% of maximal effort, 4 times for 2 min, 3 times a week) on endothelial function in hypertensive individuals; and dynamic resistance training at moderate intensity (8 exercises, 60 min/session, 3 times a week, 2×8-12 repetitions until local muscle fatigue) was examined in two studies in prehypertensive individuals. Based on the RCTs involving isometric training assessed, we can speculate that training at low intensity can improve localized endothelial function. As for dynamic resistance training, it could be an alternative to improve endothelial function in prehypertensive individuals when performed at moderate intensity.

### Aerobic training

Westhoff et al.^27^ developed a low-intensity aerobic exercise training protocol (sessions 2 times a week for 12 weeks) using upper limb cycle ergometer to assess vascular response in patients with hypertension and found no improvement in endothelium-dependent vasodilation of arm vessels. One explanation may be exercise intensity: blood lactate was 2.0 mmol/L, which is a near resting level. The association with exercise intensity is further supported by the fact that individuals with stage I-II hypertension showed improvement of endothelium-dependent vasodilation of arm vessels after aerobic training at near-maximum-intensity exercise.^28^ In addition to low-intensity exercise in this study, the use of beta-blockers may have caused systolic volume and cardiac output decrease and reduced shear stress-mediated NO release from endothelial cells, causing therefore less vasodilation.^29^

The intensity of aerobic exercise appears to influence vasomotor response in hypertensive individuals. Aerobic training for an hour on a stationary bike 3 times a week for 6 months at moderate intensity (50% HRreserve) was proven to increase plasma NO levels in hypertensive women.^30^ Molmen-Hansen et al.^28^ reported that a 3-month aerobic training improved endothelium-dependent vasodilation of arm vessels in hypertensive individuals only at high-intensities (alternating exercise at 60–70% and 90–95% HRmax). It raises the question of which other factors besides increased NO levels may contribute to the improvement of vasomotor function in response to aerobic training in hypertensive individuals.

Hypertension is associated with increased sympathetic activity that is enhanced during exercise. In normotensive individuals, exercise causes an attenuation of sympathetic activity in the active muscles with consequent local vasodilation.^31^ This late local vasodilation is parallel to the increasing intensity of muscular work, and this phenomenon involves changes in muscle metabolites and other substances to reduce vascular response to the activation of α-adrenergic receptors involved in the regulation of vascular tone.^32^ On the other hand, this mechanism is attenuated in hypertensive individuals,^31^ and along with increased arterial stiffness, it leads to reduced blood flow and shear stress during exercise.^33^ These factors may act together, precluding improvements in vasomotor capacity in hypertensive individuals following aerobic exercise in moderate or close to moderate intensity, even with preserved NO synthesis.^30^

Interval training appears to benefit vascular health in prehypertensive individuals. As it was demonstrated by Beck et al.,^34^ an exercise training program consisting of walking for 3 minutes at moderate intensity alternated with running for 2 minutes at vigorous intensity (alternating exercise at 65–85% HRmax) 3 times a week for 8 weeks may increase endothelial-dependent vasodilation in prehypertensive young.^35^

Contrary to the body of evidence on high-intensity exercise, some studies reported improvements in endothelium-dependent vasodilation of arm vessels in hypertensive elderly patients after 12 weeks of low-intensity aerobic training (blood lactate level ≤2.5 mmol/L).^36^^,^^37^ However, an important factor affecting the improvement of endothelial function following aerobic training in individuals with hypertension is endothelial dysfunction, i.e., endothelium-dependent vasodilation assessed by FMD lower than 5.5%.^38^ Thus, the variation of results from studies involving aerobic exercise at low-and moderate-intensity may also be explained by baseline endothelial dysfunction in participants.

Vascular health in hypertensive individuals in response to aerobic training may be influenced by their lipid profile. In two studies, Higashi et al demonstrated that a 3-month training consisting of unsupervised walking 5–7 times a week at moderate intensity (50% VO_2_max) for 30 minutes improved vasodilation of forearm vessels in untreated hypertensive individuals.^39^^,^^40^ Interestingly, the improvement in vasodilation of forearm vessels was negatively correlated with LDL-cholesterol levels. Thus, since hypertension is commonly associated with low HDL and high LDL levels, failure to modify the lipid profile in this population may contribute to unsatisfactory improvement in endothelial function.

Circulating levels of EMPs in peripheral blood are associated with endothelial integrity. EMPs are small membrane vesicles that are released from endothelial cells in response to cell activation, injury and apoptosis. The major cell surface markers include CD144+, CD31+/CD41-, CD31+/CD42b-, CD31+/Annexin V+ and CD62E.^41^ EMPs have been associated with the Framingham risk score,^3^ hypertension,^42^ among other conditions. While studying Afro-Americans, Feairheller et al.^43^ investigated the effects of vigorous aerobic training (up 65% VO_2_max) for 6 months.^43^ They reported that FMD increased by 60% and plasma NO levels increased by 77% along with a 50% reduction in EMP counts. However, of the 25 individuals of the sample, 10 were normotensives, 9 prehypertensives and only 7 were hypertensives, making it difficult to extrapolate the data to all three populations. It appears that exercise-induced shear stress can preserve endothelial function through a mechanism that potentiates metabolic functions of vascular cells.

The balance between endothelial injury and repair is the most significant event in the pathogenesis of atherosclerosis. EPCs play an important role in repairing injured endothelial cells and maintaining endothelial integrity. A low number of EPCs expressing the phenotype CD34+/KDR+ is predictive of cardiovascular events and death^44^ and low levels of EPCs expressing the CD34+/KDR+/CD45dim phenotype is a strong predictor of atherosclerotic disease progression.^4^ It is well established that hypertensive individuals have low numbers of functioning EPCs.^5^ In turn, aerobic training increases the levels of EPCs in patients with cardiovascular risk or established cardiovascular disease,^45^ balancing out endothelial injury and repair. However, we did not find any studies associating aerobic training and EPCs in prehypertensive and hypertensive individuals. Further investigations are required on this subject.

### Resistance training

To date, one RCT has reported the results of isometric resistance training on endothelial function in hypertensive individuals. They assessed endothelium-dependent vasodilation of arm vessels in hypertensive individuals following unilateral and bilateral isometric handgrip training.^46^ Interestingly, endothelium-dependent vasodilation improved in the trained arm only (trained arm FMD increased from 2.4 to 6.6%, p<0.001; with no change observed in untrained arm).^46^ It can thus be assumed that a greater muscle mass subjected to training is required to achieve global endothelial function benefits in this population. It is important to emphasize that most of the included articles performed the FMD technique from Corretti et al.,^47^ even those published after 2011, a period in which the technique was already updated. This allows us to question how the current technique, which is more accurate, could alter vascular results found, optimizing them.

Beck et al.^34^ examined the effects of dynamic resistance training in prehypertensive individuals. They found that one-hour training, 3 times a week for 2 months consisting of 2×8 to 12 maximum repetitions (moderate intensity) increased endothelium-dependent vasodilatation of arm vessels and reduced ET-1 levels. This same protocol was repeated to assess vascular function in the upper and lower limbs by venous occlusion plethysmography.^35^ They found improved vasodilation of forearm and leg vessels as well as improved oxidant-antioxidant balance at the end of the 2-month training. Increased endothelium-dependent vasodilation may be explained by the mechanical occlusion of vessels during exercise that causes continuous ischemia and reperfusion periods in the trained limbs, increases shear stress and leads to local endothelial adaptive changes that chronically increase vasodilatory capacity.^33^ Another possible explanation is increased blood flow to the trained muscles. This redistribution of blood flow during exercise increases systolic antegrade and diastolic retrograde blood flow that may induce increased shear stress in the vessels of the untrained limbs.^29^ Contrasting with resistance exercise, aerobic exercise continuously increases blood flow, which may lead to increased shear stress^48^ and greater exercise-induced vascular adaptations when compared to other modalities. However, improvements in endothelial function in untrained limbs appear to be similar in healthy young individuals and individuals with type 1 diabetes after an exercise session consisting of both aerobic and resistance training with similar duration, intensity and muscle groups trained.^25^ This finding raises the possibility that these variables may have impacted the results and could explain inconsistencies among the studies.

We did not find any studies involving isometric or dynamic resistance training that measured EMPs and EPCs in prehypertensive and hypertensive individuals. It requires further investigation.

The study has some limitations that need to be considered. Different aerobic training strategies (brisk walking, cycling and treadmill), participant ages and intervention times assessed in the RCTs make it difficult to infer the effect of each factor on endothelial function. Given the limited body of evidence for resistance training, further investigation is needed so we can delineate the improvement effects on endothelial function in individuals with altered blood pressure, as these are so far speculative.

## Conclusion

In the studies included in our systematic review, moderate intensity aerobic training for 30–40 minutes/session and at least 3 times a week is effective to improve endothelial function in hypertensive individuals. In prehypertensive individuals, the vigorous intensity interval aerobic training, 45 minutes/session and 3 times a week seems to be an alternative to determine vascular health benefits. As a perspective, resistance exercise training, either isometric or dynamic, could be used as a secondary strategy to improve endothelial function in individuals with altered blood pressure measurements. With regard to EPCs and EMPs data, no studies involving isometric or dynamic resistance training had measured EMPs and EPCs in prehypertensive and hypertensive individuals.
